# Cognitive impairment after post-acute COVID-19 infection: a systematic review of the literature

**DOI:** 10.1192/j.eurpsy.2023.322

**Published:** 2023-07-19

**Authors:** N. Sansone, P. Pezzella, A. Perrottelli, G. M. Giordano, E. Caporusso, L. Giuliani, P. Bucci, A. Mucci, S. Galderisi

**Affiliations:** University Of Campania “Luigi Vanvitelli”, Naples, Italy

## Abstract

**Introduction:**

After coronavirus disease 2019 (COVID-19) infection, many individuals reported neurological and psychiatric sequelae, including cognitive impairment, even several months after the acute infection.

**Objectives:**

The present study aims to provide a critical overview of the literature on the relationships between post-acute COVID-19 infection and cognitive impairment, highlighting limitations and confounding factors.

**Methods:**

A systematic search of articles published from January 1st, 2020, to July 1st, 2022 was performed in Pubmed/Medline. We followed the Preferred Reporting Items for Systematic Reviews and Meta-Analyses (PRISMA) guidelines.

**Results:**

Only studies using validated instruments for the assessment of cognitive impairment were included. Out of 5478 screened records, 72 studies met inclusion criteria. Time of patients’ assessment varied from 4 weeks to 12 months after the infection. The available evidence revealed the presence of impairment in executive functions, attention and memory in subjects recovered from COVID-19. However, several limitations of the literature reviewed should be highlighted: most studies were performed on small samples, not stratified by severity of disease and age, used a cross-sectional or a short-term longitudinal design, and provided a limited assessment of the different cognitive domains. Few studies investigated neurobiological correlates of cognitive deficits in individuals recovered from COVID-19.

**Conclusions:**

Based on the literature reviewed, it is difficult, to date, to draw conclusions about the relationships between COVID-19 infection and cognitive impairment. Therefore, further studies with an adequate methodological design are needed in order to better understand these relationships, identify neurobiological correlates of COVID-related cognitive deficits and evaluate their course over time. Enhancing the knowledge on this topic could favor the development of effective therapeutic strategies for cognitive deficits in individuals recovered from COVID-19.

**Disclosure of Interest:**

None Declared

